# Virulence and antimicrobial resistance in *Salmonella enterica* serovar Typhimurium: a One Health perspective on therapeutic and vaccine targets

**DOI:** 10.3389/fmicb.2026.1851580

**Published:** 2026-06-11

**Authors:** Mohammed Naveez Valathoor, Anand Prem Rajan

**Affiliations:** Department of Bio-Medical Sciences, School of Biosciences and Technology, Vellore Institute of Technology, Vellore, Tamil Nadu, India

**Keywords:** antimicrobial resistance, One Health, *Salmonella enterica* serovar Typhimurium, vaccine development, virulence factors

## Abstract

*Salmonella enterica* serovar Typhimurium (*S. Typhimurium*) is a major non-typhoidal *Salmonella serovar* associated with a substantial global burden of foodborne and invasive infections. Its transmission across human, animal, food, and environmental interfaces highlights its significance its relevance within a One Health framework. The pathogenicity of *S. Typhimurium* is mediated by multiple virulence determinants, including *Salmonella* pathogenicity islands (SPI-1 and SPI-2), type III secretion systems (T3SS), fimbrial adhesins, and biofilm formation, which contribute to host cell invasion, intracellular survival, and persistence. The increasing prevalence of antimicrobial resistance (AMR) in *S. Typhimurium* is driven by horizontal gene transfer and chromosomal mutations, involving resistance determinants such as β-lactamase genes (*blaCTX-M, blaVIM*), plasmid-mediated quinolone resistance genes (*qnr*), colistin resistance genes (*mcr*), and mutations in target genes (e.g., *gyrA, gyrB*). These mechanisms have reduced the effectiveness of commonly used antibiotics and contributed to the emergence of multidrug-resistant strains. This review synthesizes current knowledge on the epidemiology, transmission dynamics, virulence mechanisms, and AMR profiles of *S. Typhimurium*, including global burden indicators such as Disability-Adjusted Life Years (DALYs) and region-specific trends in India. Current therapeutic approaches and vaccine candidates are also evaluated, highlighting existing limitations and research gaps. Emphasis is placed on the interaction between virulence and AMR and the identification of conserved molecular targets to support the development of effective interventions within a One Health framework.

## Background

*Salmonella* is a genus of Gram-negative, facultative anaerobic, rod-shaped bacteria belonging to the family *Enterobacteriaceae*. The genus includes more than 2,500 serovars, classified based on somatic (O) and flagellar (H) antigens (Grimont and Weill, 2007; Brenner et al., 2000). Among these, *Salmonella enterica* subspecies *enterica* serovar Typhimurium (*S. Typhimurium*) is one of the most clinically and epidemiologically significant non-typhoidal *Salmonella* (NTS) serovars globally (Gal-Mor et al., 2014). Unlike typhoidal serovars (*S. typhi, S. paratyphi*), *S. Typhimurium* has a broad host range and infects humans, poultry, cattle, swine, rodents, and companion animals, making it a major zoonotic and foodborne pathogen (Bharathan and Srikumar, 2025). The ability of *S. Typhimurium* to cause disease is driven by an extensive arsenal of virulence determinants encoded in *Salmonella* pathogenicity islands (SPIs), plasmids, prophages, fimbrial clusters, and two-component regulatory systems such as *PhoP/PhoQ* and *HilA/HilD*. These virulence factors facilitate adhesion, invasion, intracellular survival, biofilm formation, immune evasion, and systemic spread in susceptible hosts (Fàbrega and Vila, 2013; Lou et al., 2019). With the rapid expansion of genomic data, *in silico* approaches have become indispensable for identifying, annotating, and characterizing virulence genes in *S. Typhimurium*.

## Global epidemiology

**Figure 1:**
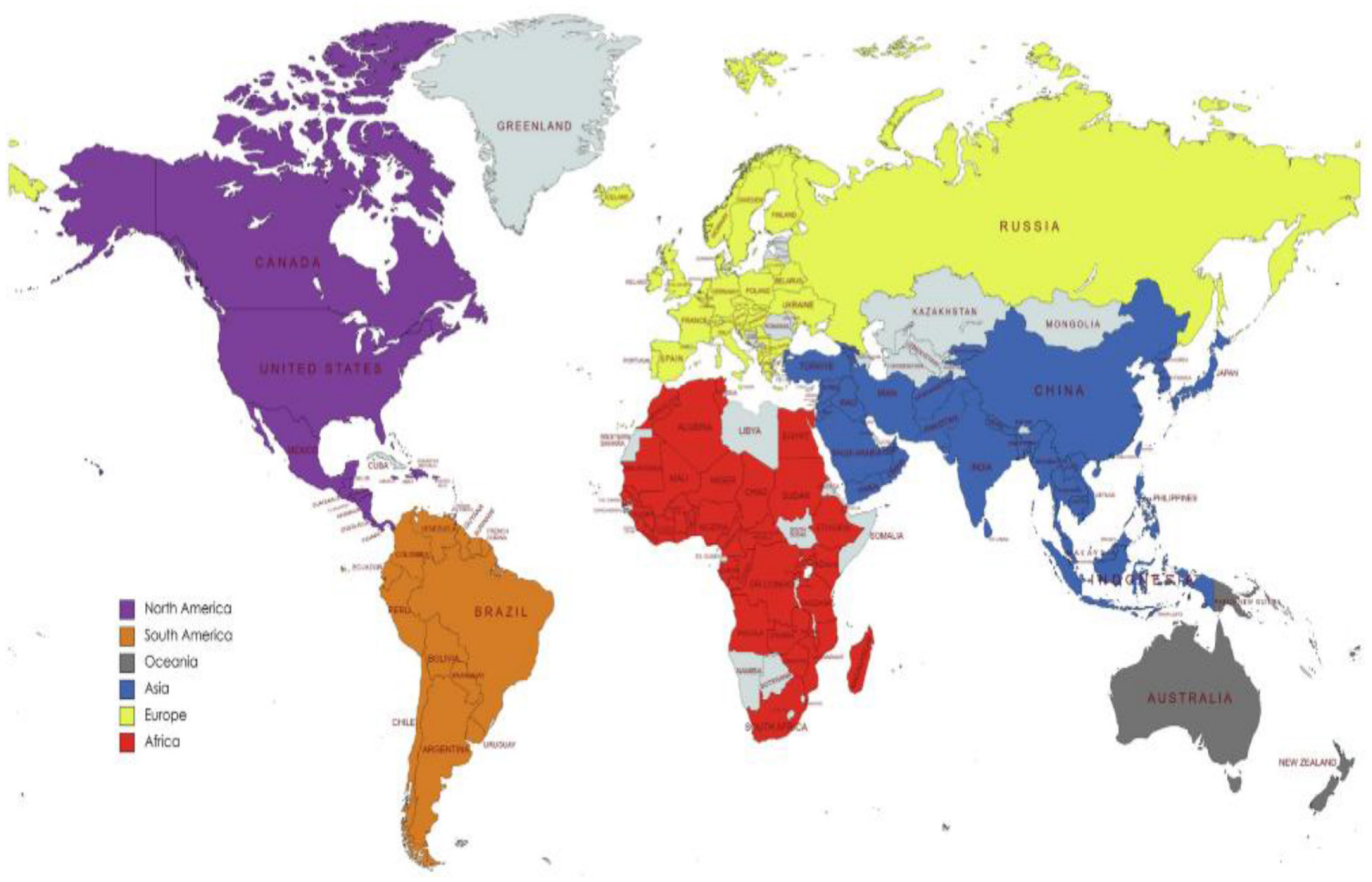
Global distribution of antimicrobial-resistant *Salmonella enterica* serovar Typhimurium. Geographic variation in antimicrobial resistance (AMR) prevalence across regions, highlighting the distribution of multidrug-resistant (MDR), fluoroquinolone-resistant, and ESBL-producing strains. This figure supports trends discussed in the Global Epidemiology section. *Source: Adapted from* Wang et al. (2025).

Non-typhoidal *Salmonella* (NTS) is a major contributor to the global burden of foodborne infectious diseases (Figure 1). Estimates from the Global Burden of Disease (GBD) 2017 study, generated using systematic surveillance data, hospital records, literature reviews, and statistical disease-burden modeling approaches, indicate that NTS causes approximately 93.8 million enteric infections and nearly 155,000 deaths annually worldwide (Stanaway et al., 2019). Among NTS serovars, *Salmonella enterica* serovar Typhimurium is consistently one of the most frequently isolated from both human and animal infections across continents. Genomic studies have revealed distinct population structures within this serovar, including globally distributed ST19 and the invasive ST313 lineage, the latter strongly associated with bloodstream infections in sub-Saharan Africa (Kumwenda et al., 2024; Van Puyvelde et al., 2023). Beyond gastroenteritis, invasive non-typhoidal *Salmonella* (iNTS) disease represents a critical but often under recognized component of the global burden. According to the Global Burden of Disease (GBD) 2017 estimates, iNTS accounted for approximately 535,000 cases and 77,500 deaths in 2017, disproportionately affecting children under 5 years, elderly individuals, and immunocompromised populations, particularly those with Human Immunodeficiency Virus (HIV) infection. Importantly, iNTS disease contributed an estimated 4.26 million disability-adjusted life years (DALYs) globally, with the burden largely driven by years of life lost (YLLs), reflecting its high case fatality and acute severity despite lower incidence compared to enterocolitis (Stanaway et al., 2019). Global antimicrobial resistance (AMR) surveillance studies have highlighted the growing public health concern associated with *S*. Typhimurium, particularly due to the increasing emergence and dissemination of fluoroquinolone-resistant, cephalosporin-resistant, and multidrug-resistant (MDR) strains, which significantly compromise treatment efficacy and limit therapeutic options (Hendriksen et al., 2019). Reflecting this growing threat, the World Health Organization (WHO) has categorized fluoroquinolone-resistant non-typhoidal *Salmonella*, including *S. Typhimurium*, as a High Priority Pathogen in the 2024 Bacterial Priority Pathogens List (World Health Organization, 2024). Together, these trends highlight the significant and evolving global epidemiology of *S. Typhimurium*, emphasizing the need for strengthened surveillance, antimicrobial stewardship, and effective vaccine development strategies.

## Epidemiology in India

In India, *S. Typhimurium* is increasingly recognized in both clinical and veterinary surveillance. Hospital-based studies report its frequent isolation from diarrhoeal cases and bloodstream infections, with some regions documenting its predominance among extra-intestinal NTS infections (Kumar et al., 2022). NTS, including *S. Typhimurium*, has been isolated from poultry, cattle, pigs, seafood, and retail food samples across India, reflecting widespread contamination in the food chain (Bangera et al., 2019; Dudhane et al., 2023). The Indian council of medical research (ICMR) AMR surveillance has identified increasing rates of resistance to ciprofloxacin and rising prevalence of extended-spectrum β-lactamases (ESBL) producing NTS strains (Indian Council of Medical Research (ICMR), 2023). Additionally, the extensive use of antibiotics in livestock production, along with inadequate biosecurity measures and poor environmental sanitation, has been recognized as a major factor contributing to the emergence and dissemination of resistant *S*. Typhimurium strains in the country (Geyi et al., 2024; Kumar et al., 2022). In India, the emergence of antibiotic-resistant *S. Typhimurium* strains from food sources has important clinical implications. Contaminated poultry, meat, seafood, and fresh produce act as major reservoirs of multidrug-resistant (MDR) strains, which are increasingly associated with human infections (Bangera et al., 2019; Dudhane et al., 2023). Infections caused by these resistant strains are linked to prolonged illness, higher rates of hospitalization, and an increased risk of invasive disease, particularly in vulnerable populations. The presence of resistance to commonly used antibiotics such as fluoroquinolones and third-generation cephalosporins further complicates treatment, often leading to delayed therapeutic response or treatment failure. In some cases, this necessitates the use of last-resort antibiotics, increasing both treatment cost and the risk of further resistance development. Additionally, foodborne transmission of resistant strains contributes to the silent dissemination of AMR within the community, even among individuals with no prior antibiotic exposure. These findings highlight the significant public health impact of foodborne antimicrobial-resistant *S. Typhimurium* in India and reinforce the need for integrated surveillance, improved food safety practices, and stricter regulation of antibiotic use in livestock production.

## Transmission

**Figure 2:**
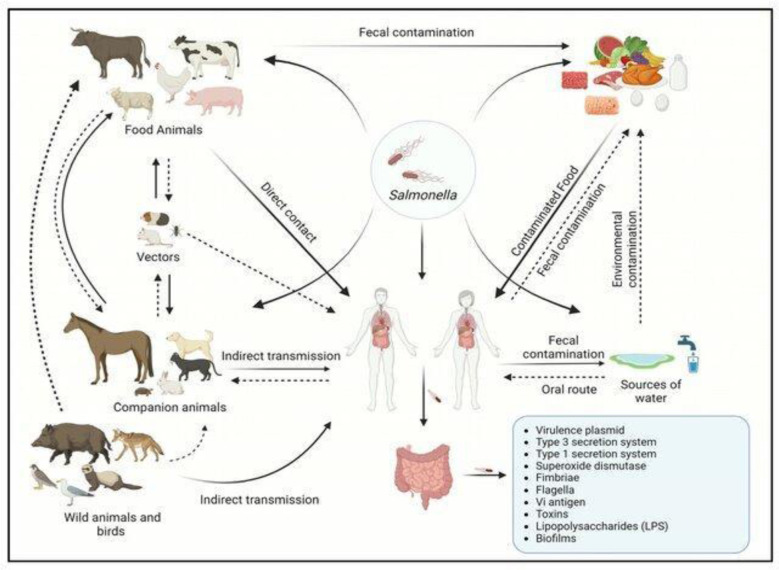
One Health transmission cycle of *Salmonella enterica* serovar Typhimurium. Schematic representation of transmission across humans, animals, food systems, and the environment. Arrows indicate major routes including foodborne, zoonotic, and environmental pathways, along with key virulence factors such as T3SS, fimbriae, and biofilm formation that facilitate infection and persistence. *Source: Adapted from* Minho (2024).

The transmission of *Salmonella enterica* serovar Typhimurium involves interconnected pathways spanning humans, animals, food systems, and environmental reservoirs (Figure 2). Foodborne exposure represents the predominant route globally, with non-typhoidal *Salmonella* causing an estimated ~93 million infections annually, and *S. Typhimurium* among the most frequently reported serovars in both clinical and food sources (Majowicz et al., 2010; World Health Organization, 2024). Common vehicles include poultry, eggs, pork, beef, milk, seafood, and fresh produce (Oludairo et al., 2023). Contamination can occur throughout the farm-to-fork continuum, beginning with intestinal colonization and fecal shedding in asymptomatic animals, followed by dissemination during slaughter, processing, retail handling, and domestic food preparation (Teklemariam et al., 2023). Reported contamination rates in poultry products, often ranging from 20 to 70%, highlight the efficiency with which the organism is maintained within food production systems. Within poultry production, both vertical and horizontal transmission contribute to persistence. Vertical transmission occurs through contamination of eggs from infected breeder flocks, whereas horizontal spread is mediated by contaminated litter, feed, water, and equipment surfaces. Rodents, flies, and farm personnel act as mechanical vectors that sustain environmental circulation (Bharathan and Srikumar, 2025). In addition, waterborne transmission remains relevant in settings with inadequate sanitation infrastructure. Contaminated irrigation water introduces *Salmonella* onto fresh produce, including leafy vegetables and tomatoes, which are frequently implicated in outbreaks (Reyes et al., 2025). Zoonotic transmission further reflects the broad host range of *S. Typhimurium*, with infections linked to direct contact with livestock, reptiles, amphibians, and wildlife. Rodents, in particular, function as important reservoirs in poultry farms and storage environments (Liu et al., 2022).

Beyond these transmission routes, environmental persistence plays a critical role in sustaining contamination cycles. Persistence within these environments is reinforced by the capacity of *S. Typhimurium* to form biofilms on both biotic and abiotic surfaces, including stainless steel, plastic, and rubber used in food processing facilities. Biofilms consist of structured bacterial communities embedded in an extracellular polymeric substance (EPS) matrix composed of polysaccharides, proteins, and extracellular Deoxyribonucleic Acid (DNA). This matrix limits the penetration and activity of disinfectants such as chlorine and quaternary ammonium compounds, resulting in reduced susceptibility compared to planktonic cells. Biofilm-associated bacteria may exhibit substantially increased tolerance to antimicrobial agents and environmental stress, enabling long-term survival under adverse conditions. In addition, the close cellular proximity within biofilms facilitates horizontal gene transfer, supporting the maintenance and dissemination of resistance determinants. Persistent biofilms have been associated with recurrent contamination in food processing environments despite routine sanitation (Kebenei et al., 2025). Human-to-human transmission is less common but can occur through the fecal–oral route, particularly in institutional settings such as hospitals, childcare centers, and nursing homes (Johnston et al., 2025). These events are typically linked to inadequate hygiene and high organism load. Taken together, these transmission routes illustrate the ability of *S. Typhimurium* to persist and disseminate across multiple ecological niches. Effective control requires coordinated measures addressing animal reservoirs, food production practices, environmental contamination, and hygiene, consistent with a One Health framework.

## Clinical manifestations

Non-typhoidal *Salmonella* infections caused by *S. Typhimurium* most commonly present as acute gastroenteritis, characterized by the sudden onset of diarrhea, vomiting, fever, abdominal cramps, and systemic malaise. Symptoms typically appear 6 to 72 h after ingestion of contaminated food or water and generally resolve within 3–7 days in immunocompetent individuals (Acheson and Hohmann, 2001). However, the clinical spectrum is highly variable and strongly influenced by host immunity, virulence determinants, and infectious dose. In susceptible populations including infants, the elderly, and immunocompromised persons *S. Typhimurium* can breach intestinal epithelial barriers and disseminate via macrophages to cause invasive non-typhoidal *Salmonella* (iNTS) disease, manifesting as bacteremia, sepsis, meningitis, osteomyelitis, or focal organ abscesses (Feasey et al., 2012). The pathogenicity is largely mediated by SPI-1 and SPI-2 encoded Type III Secretion System (T3SS) effectors that orchestrate epithelial invasion and intracellular survival, enabling persistence within macrophages and contributing to recurrent or chronic infections in some individuals (Srividhya et al., 2025). Severe outcomes are frequently reported in regions with high HIV prevalence, malnutrition, or comorbidities, highlighting the public health importance of this serovar. While most gastrointestinal infections are self-limiting, the potential for invasive disease and mortality underscores the need for early recognition and appropriate clinical management.

## Molecular pathogenesis

The pathogenic prowess of *S. Typhimurium* is not a random occurrence but the product of a highly sophisticated and tightly regulated molecular arsenal. The majority of its key virulence determinants are not scattered randomly throughout its chromosome but are consolidated within large, mobile genetic loci known as *Salmonella* pathogenicity islands. These islands, often acquired through horizontal gene transfer, function as modular pathogenic toolkits, encoding clusters of genes that work in concert to orchestrate the complex processes of host invasion, intracellular survival, and immune evasion (Eng et al., 2015; Lou et al., 2019). The major virulence factors associated with these processes are summarized in Table 1.

**Table 1:** Summary of *S. Typhimurium* virulence factors and functional roles.

Virulence factor	Description and function	Genes/proteins involved	References
Type III secretion systems	Molecular injectisomes that deliver effector proteins into host cells to mediate invasion and intracellular survival	invA, sipA, sipB, sopE, sopB, sopD, ssaV, sseB, sseC, sifA	Worley, 2025
*Salmonella* pathogenicity islands	Genomic regions encoding virulence factors including T3SS, adherence proteins, and regulatory elements	SPI-1 to SPI-5 clusters; key genes: inv, prg, ssa, sii, sop	Lou et al., 2019
Adhesins and fimbriae	Surface structures aiding bacterial adhesion to host cells and biofilm formation	FimA, FimH, LpfA	Zeiner et al., 2012
Lipopolysaccharide	Outer membrane component involved in immune evasion and triggering host inflammatory responses	Genes involved in O-antigen and lipid A biosynthesis—rfa, rfb clusters	Magaña et al., 2023
Capsular polysaccharide (Vi antigen)	Capsule-like polymer providing resistance to phagocytosis and complement-mediated killing	viaB operon	Wilson et al., 2011
Flagella	Enables motility and chemotaxis; also recognized by host immune system	fliC, fljB	Xia et al., 2025
Toxins	Secreted proteins causing host cell damage or immune modulation	SpvB genes	Dong et al., 2022
Effector proteins	Bacterial-secreted proteins that manipulate host cell functions	SopE2, SptP	Jamali et al., 2025
Regulatory systems	Two-component systems modulating expression of virulence genes in response to host environment	PhoP/PhoQ, SsrAB, RcsB	Shaw et al., 2022
Biofilm formation	Multicellular communities enabling persistence and resistance to environmental stressors	csgA, bcs operon, adrA	Das and Behera, 2025
Iron acquisition systems	Mechanisms to scavenge iron essential for bacterial growth	SitABCD, FeoABC, IroN, FepABC	Domínguez-Acuña and García-Del, 2022

## *Salmonella* pathogenicity islands (SPIs)

**Figure 3:**
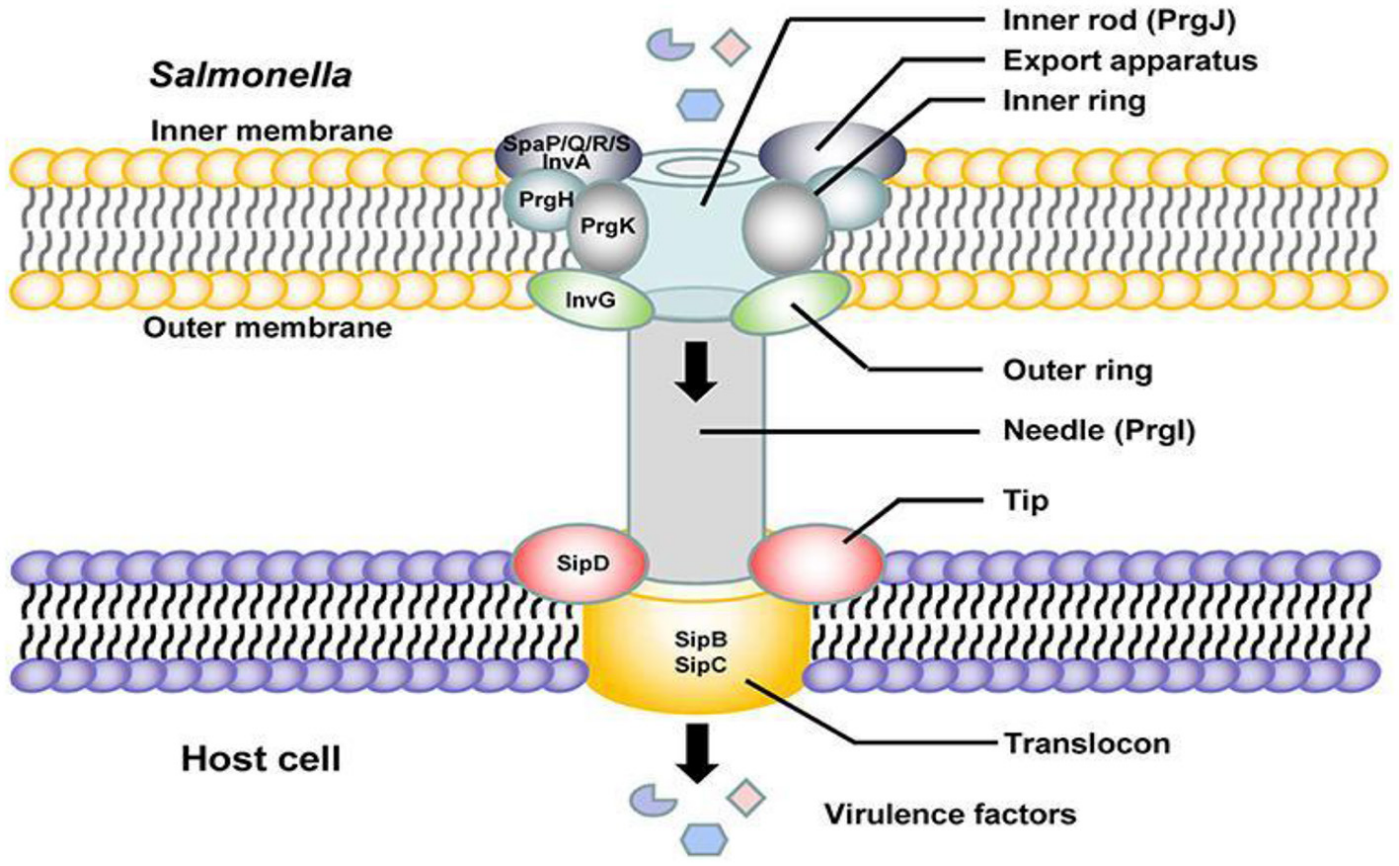
SPI-1–mediated invasion of epithelial cells by *Salmonella enterica* serovar Typhimurium via T3SS-1, involving effector-driven actin rearrangement, membrane ruffling, and bacterial internalization. *Source: Adapted from* Lou et al. (2019).

*Salmonella* pathogenicity islands represent the central virulence determinants of *S. Typhimurium*, with SPI-1 and SPI-2 playing dominant roles during infection. While multiple SPIs (SPI-1 to SPI-5) have been identified, the early and critical stages of infection are primarily governed by these two systems. The hallmark of *Salmonella* virulence is its ability to actively invade non-phagocytic intestinal epithelial cells through the Type III secretion system encoded on SPI-1 (T3SS-1). This system functions as a molecular injectisome that delivers effector proteins directly into host cells upon contact (Figure 3). The expression of T3SS-1 is tightly regulated by transcription factors such as HilA, which respond to environmental cues including low oxygen levels and high osmolarity within the intestinal lumen.

Effector proteins including SopE, SopE2, SopB, SipA, and SipC orchestrate host cytoskeletal rearrangements. SopE and SopE2 activate Rho-family Guanosine Triphosphate (GTP)ases such as Cdc42 and Rac1, inducing actin polymerization, while SipA stabilizes actin filaments. SopB modifies phosphoinositide signaling, leading to membrane destabilization. These coordinated processes result in membrane ruffling and bacterial internalization via macropinocytosis.

Following internalization, *S. Typhimurium* resides within the *Salmonella*-containing vacuole (SCV), where SPI-2 becomes functionally active. The SPI-2-encoded T3SS-2 is essential for intracellular survival and systemic dissemination. It translocates effector proteins such as SseF, SseG, and SifA, which interfere with host intracellular trafficking pathways and prevent lysosomal fusion. SifA also mediates the formation of *Salmonella*-induced filaments, which are crucial for maintaining vacuolar integrity and facilitating nutrient acquisition. These adaptations enable bacterial replication within macrophages and dissemination to systemic organs such as the liver and spleen (Lou et al., 2019; Matsuda et al., 2019).

## Fimbrial clusters and adhesion mechanisms

The infection process is initiated by adhesion to the intestinal epithelium, a critical step for establishing colonization. *S. Typhimurium* expresses multiple fimbrial structures that facilitate host cell attachment and environmental persistence. Type 1 fimbriae mediate binding to mannose-containing glycoproteins on epithelial cells via the *FimH* adhesin, enabling stable colonization (Cheng and Wiedmann, 2021; Lindberg et al., 2025).

In addition, long polar fimbriae contribute to adhesion to Peyer's patches and intestinal M cells, enhancing bacterial translocation across the intestinal barrier. Curli fimbriae, composed of amyloid fibers, play a dual role in adhesion and biofilm formation. These structures promote surface attachment and multicellular community formation, thereby enhancing resistance to environmental stress and host immune defenses. The coordinated expression of fimbrial operons allows *Salmonella* to adapt to different host environments and stages of infection.

## Two-component regulatory systems and additional virulence determinants

Two-component regulatory systems play a crucial role in controlling *Salmonella* virulence by enabling the bacterium to sense and respond to environmental stimuli. These systems typically consist of a membrane-bound sensor kinase and a cytoplasmic response regulator that modulate gene expression.

The *PhoP/PhoQ* system is activated under conditions such as low magnesium concentration and exposure to antimicrobial peptides, leading to the expression of genes involved in resistance to host defenses and intracellular survival. The *SsrA/SsrB* (SsrAB) system specifically regulates SPI-2 gene expression and is activated within the acidic and nutrient-limited environment of the SCV. Additionally, the Rcs phosphorelay system contributes to capsule production, biofilm formation, and stress response.

Beyond these regulatory mechanisms, virulence plasmids also contribute significantly to pathogenicity. The spv operon enhances intracellular replication and systemic spread by modulating host immune responses. Prophage-encoded factors such as superoxide dismutases (SodCI and SodCII) provide protection against oxidative stress within host phagocytes. This complex and coordinated virulence architecture, summarized in Table 1, underscores the sophisticated strategies employed by *S. Typhimurium* to establish infection and evade host defenses (Azimi et al., 2020).

## Rise of antimicrobial resistance

**Figure 4:**
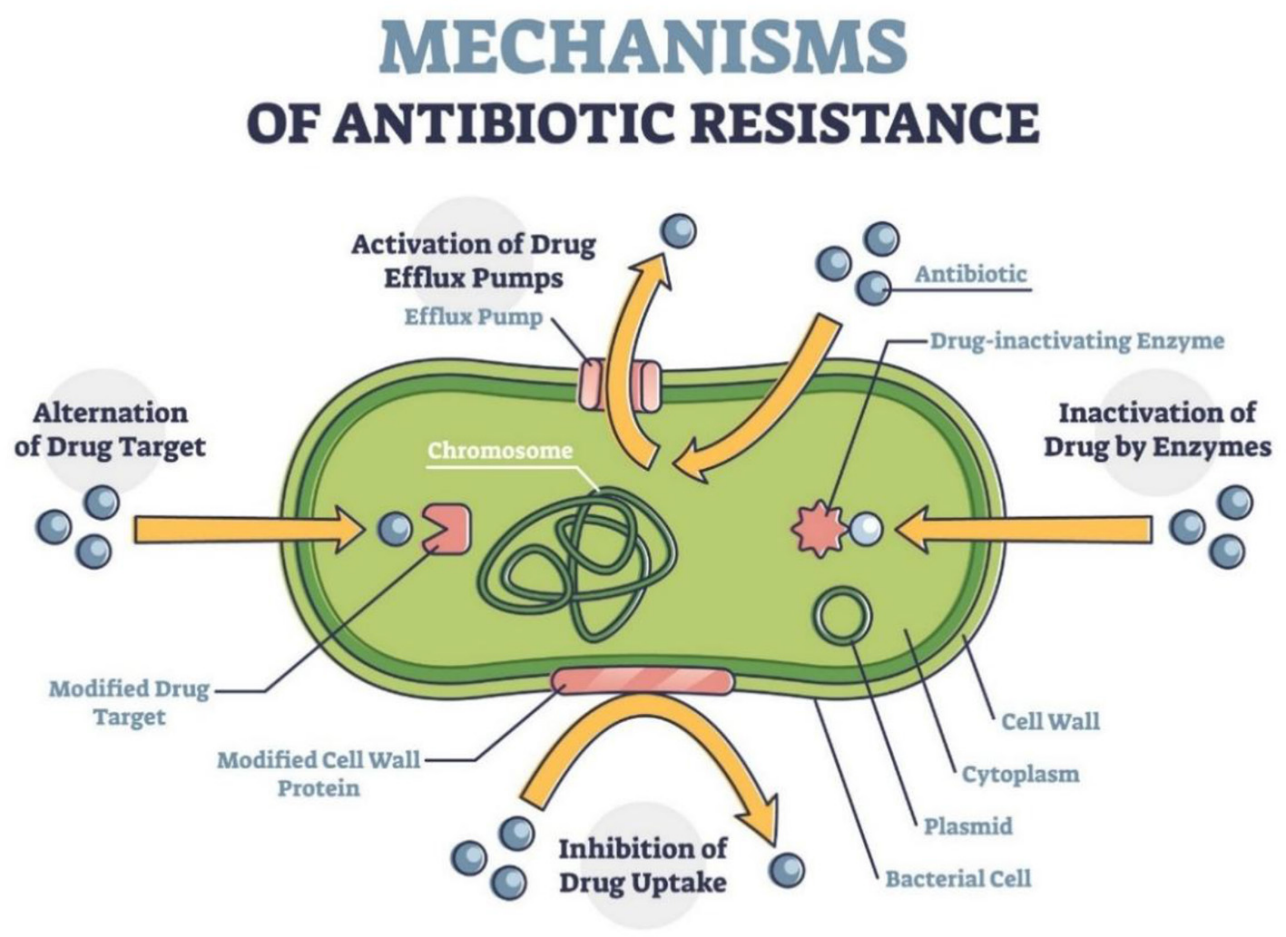
Molecular mechanisms of antimicrobial resistance in *Salmonella enterica* serovar Typhimurium. Overview of resistance mechanisms including enzymatic degradation (β-lactamases), target modification (gyrA/parC mutations), reduced membrane permeability, efflux pumps, and horizontal gene transfer via mobile genetic elements, as discussed in the AMR section. *Source: Adapted from* Teklemariam et al. (2023).

The rise of antimicrobial resistance (AMR) in *Salmonella enterica* serovar Typhimurium is driven by a combination of selective antibiotic pressure, extensive antimicrobial use in animal agriculture, and the efficient spread of resistance determinants through horizontal gene transfer (Figure 4). Over time, this serovar has accumulated resistance to multiple antibiotic classes, reducing treatment options and contributing to more severe and prolonged infections (Wang et al., 2025; Hendriksen et al., 2019). Resistance in *S. Typhimurium* is mediated through several well-characterized mechanisms. β-lactam resistance is primarily associated with extended-spectrum β-lactamases (ESBLs), including genes such as *blaCTX-M, blaTEM*, and *blaSHV*, which hydrolyse third-generation cephalosporins (Edoo et al., 2017; Geyi et al., 2024). Resistance to carbapenems, although less frequently reported, has been linked to metallo-β-lactamase genes such as *blaNDM* and *blaVIM*. Fluoroquinolone resistance arises through both plasmid-mediated genes, including *qnr*, and chromosomal mutations in quinolone resistance-determining regions (QRDRs), particularly in *gyrA, gyrB*, and *parC*, which reduce drug binding affinity (Mohammed et al., 2021). In addition, plasmid-mediated colistin resistance genes such as *mcr-1* have emerged as an important concern due to the limited availability of alternative therapies. The dissemination of these resistance determinants is strongly linked to horizontal gene transfer. Mobile genetic elements, including conjugative plasmids, class 1 integrons, and transposons such as Tn21, facilitate the capture and spread of resistance gene cassettes across bacterial populations (Tokuda and Shintani, 2024). These elements often carry multiple resistance genes, promoting the emergence of multidrug-resistant (MDR) strains and, in some cases, co-localization with virulence-associated genes.

In addition to gene acquisition, physiological mechanisms also contribute to resistance. Alterations in outer membrane porins reduce antibiotic uptake, while active efflux pumps expel antimicrobial agents from the bacterial cell, lowering intracellular drug concentrations (Zhou et al., 2023; Alenazy, 2022). Biofilm formation further enhances resistance by limiting antibiotic penetration and promoting bacterial persistence. The extracellular polymeric substance (EPS) matrix, composed of polysaccharides, proteins, and extracellular DNA, acts as a diffusion barrier and creates microenvironments that support metabolically inactive cells. As a result, biofilm-associated bacteria can exhibit up to 1,000-fold higher tolerance to antimicrobial agents compared to planktonic cells (Uruén et al., 2020). At the global level, the World Health Organization has classified fluoroquinolone-resistant non-typhoidal *Salmonella* as a high-priority pathogen, reflecting its growing clinical importance (World Health Organization, 2024). In India, surveillance data from the Indian Council of Medical Research (ICMR) indicate increasing resistance to fluoroquinolones and a rising prevalence of ESBL-producing isolates in both clinical and food-associated *Salmonella* strains (Geyi et al., 2024; Srividhya et al., 2025). Overall, AMR in *S. Typhimurium* is driven by the combined effects of horizontal gene transfer, chromosomal mutation, and biofilm-associated tolerance, highlighting the need for integrated surveillance and targeted intervention strategies within a One Health framework.

## Current treatments

Treatment of *S. Typhimurium* infections requires a balanced approach that considers disease severity, host immune status, and emerging resistance patterns. For uncomplicated gastroenteritis, antimicrobial therapy is generally discouraged since it does not significantly reduce illness duration (typically 3–7 days) and may prolong bacterial shedding by up to 2–3 weeks or promote resistance development (Alemnew et al., 2024). Supportive management, including oral rehydration, electrolyte replacement, antipyretic therapy, and nutritional support, remains the mainstay. However, in patients at increased risk of severe disease, including infants, elderly individuals, pregnant women, and immunocompromised hosts, antibiotics become essential (Clinical Overview of Salmonellosis, 2024). For systemic or invasive infections, fluoroquinolones such as ciprofloxacin have traditionally been first-line therapy due to their high intracellular penetration and activity against *Salmonella* within macrophages. However, rising fluoroquinolone resistance, with reduced susceptibility reported in approximately 25%−40% of isolates in South Asia, has reduced therapeutic effectiveness (Asghar et al., 2024). Third-generation cephalosporins such as ceftriaxone are widely used, particularly in pediatric populations. However, the emergence of extended-spectrum β-lactamase (ESBL)-producing strains, with reported prevalence ranging from 10 to 20% in clinical isolates, has further limited treatment options. Azithromycin has gained prominence as an alternative therapy, with clinical success rates of approximately 80%−90% in uncomplicated and moderately severe infections, especially in regions with high fluoroquinolone resistance (Asghar et al., 2024; Shane et al., 2017). The therapeutic landscape is increasingly complicated by multidrug-resistant (MDR) strains. In severe cases involving ESBL-producing isolates, carbapenems may be required, although their use is tightly restricted to minimize resistance emergence. Adjunctive therapies such as zinc supplementation in children and probiotics may support recovery but do not replace antibiotic therapy. Given the escalating AMR crisis, research into novel treatment modalities has intensified. Promising candidates include bacteriophage therapy, antimicrobial peptides, quorum-sensing inhibitors, Clustered Regularly Interspaced Short Palindromic Repeats (CRISPR)-based antimicrobials, and anti-virulence compounds targeting T3SS effectors. Preclinical studies have reported reductions in bacterial load of approximately 2–4 log units with bacteriophage therapy and significant attenuation of virulence through T3SS inhibition (Venkataraman et al., 2025). However, their translation to clinical practice requires validation through large-scale clinical trials.

## Current vaccines

Despite the significant global burden of non-typhoidal *Salmonella*, including *S. Typhimurium*, no licensed vaccine is currently available for human use, making vaccine development a critical public health priority. This is largely due to substantial genetic and antigenic diversity among NTS serovars, their broad host range, and the lack of clearly defined correlates of protective immunity (MacLennan, 2024). Immunity to NTS is multifactorial, involving both cell-mediated and humoral responses. However, reinfections and persistent intestinal carriage indicate that natural immunity is often incomplete or short-lived. Experimental vaccine platforms being explored include live-attenuated, inactivated, subunit, outer membrane vesicle (OMV), and generalized modules for membrane antigens (GMMA)-based vaccines, as well as polysaccharide–protein conjugates. Recombinant attenuated *Salmonella* vaccines, with deletions in genes such as *aroA, phoP/phoQ*, or *ssaV*, have demonstrated strong immunogenicity, with approximately 10–100-fold increases in antigen-specific IgG responses in preclinical studies (Chagas et al., 2024). However, balancing safety and immunogenicity remains a challenge, particularly in immunocompromised populations. Radiation-mutagenized vaccines, such as ATOMSal-L6, have demonstrated protective efficacy of approximately 70%−90% in murine and porcine models, with significant reductions in bacterial colonization and systemic dissemination (Ji et al., 2022). Vesicle-based vaccines, including OMV and GMMA formulations, have progressed to Phase I/II trials. These candidates have demonstrated approximately 4–10-fold increases in anti-O-antigen IgG titres, along with favorable safety profiles and scalability (Hanumunthadu et al., 2023; Skidmore et al., 2023). Subunit vaccines, particularly O-antigen polysaccharide conjugates (e.g., CRM197-based), have shown seroconversion rates exceeding 80% in preclinical studies. However, achieving broad protection across multiple NTS serovars remains a challenge. In contrast, veterinary vaccines such as Nobilis^®^ Salenvac T (Merck Animal Health in the United States and Canada), Poulvac^®^ ST (Zoetis), and BIOSUIS SALM^®^ (Bioveta, a.s) have demonstrated 60%−80% reductions in intestinal colonization and fecal shedding, thereby limiting transmission within animal populations (Siddique et al., 2024). However, antigenic differences between veterinary strains and human isolates limit their applicability for human use. Human vaccine development continues to face challenges, including antigenic diversity, lack of defined correlates of protection, the need for durable mucosal immunity, and safety concerns in high-risk populations. Identifying conserved virulence factors through integrative and immunoinformatics approaches may support the development of broadly protective next-generation vaccines.

## Rationale for *in silico* analysis of virulence factors

*In silico* analysis provides rapid, cost-effective insights into pathogen virulence, enabling the identification of virulence genes, genomic islands, secretion system components, and protein structure function predictions. Computational genomics allows comparative analyses between global and Indian isolates, identification of conserved virulence determinants, and prediction of potential drug or vaccine targets. As large volumes of genomic data continue to accumulate, food studies are invaluable for understanding the molecular basis of *S. Typhimurium* pathogenicity and AMR virulence interactions.

## Research gaps

Although considerable progress has been made in understanding *Salmonella enterica* serovar Typhimurium biology, several significant research gaps hinder effective disease control, therapeutic innovation, and vaccine development. Foremost among these gaps is the limited comprehensive computational characterization of virulence factors, especially for Indian *S. Typhimurium* isolates, which remain underrepresented in global genomic repositories such as EnteroBase and National Center for Biotechnology Information (NCBI) Assembly databases. This restricts the ability to conduct comparative virulome analyses, identify population-specific adaptations, or correlate virulence profiles with antimicrobial resistance trends at a national scale. Another major gap involves the poorly understood relationship between virulence determinants and AMR genes, particularly their frequent co-localization on mobile genetic elements. Many plasmids in *S. Typhimurium* carry virulence genes (e.g., *spvB, spvC*) alongside ESBL genes, yet the evolutionary and functional implications of this co-selection remain inadequately studied. Understanding these relationships is essential for predicting emergence of high-risk clones with combined virulence-resistance phenotypes. A third gap concerns the insufficient integration of One-Health-based genomic surveillance, linking human, animal, food, and environmental isolates. Studies in India are often fragmented, region-specific, or limited to hospital isolates, creating an incomplete picture of transmission dynamics. Large-scale analyses linking genomic data with epidemiological metadata are rarely conducted, restricting identification of dominant clones, zoonotic transmission pathways, and environmental reservoirs. In the context of vaccine development, there is a shortage of studies identifying and validating conserved, surface-exposed, immunogenic virulence factors that could serve as universal vaccine targets across diverse *S. Typhimurium* lineages. Existing data often focus on known SPI-encoded effectors, but the potential roles of fimbrial operons, flagellar antigens, outer membrane proteins, and metabolic virulence factors remain underexplored.

## Conclusion

*Salmonella enterica* serovar Typhimurium remains a major contributor to the global burden of non-typhoidal salmonellosis globally, with a substantial burden in India driven by foodborne transmission and increasing antimicrobial resistance (AMR). Multidrug resistance (MDR) rates frequently exceed 30% in clinical and food-associated isolates, including resistance to first-line antibiotics such as ampicillin, chloramphenicol, and trimethoprim–sulfamethoxazole, along with reduced susceptibility to fluoroquinolones and third-generation cephalosporins. Its pathogenicity is mediated by key virulence determinants, including Salmonella pathogenicity islands (SPIs), type III secretion systems, and biofilm formation, which enhance host invasion, persistence, and environmental survival.

The convergence of virulence and resistance traits, often associated with mobile genetic elements, underscores its significance as a One Health pathogen with transmission across human, animal, food, and environmental interfaces. Clinically, infections range from self-limiting gastroenteritis to invasive disease in high-risk populations, with treatment options increasingly limited by resistance trends. Despite advances in genomic surveillance, critical gaps remain in linking virulence profiles with AMR patterns and clinical outcomes. Addressing these challenges will require integrated genomic and epidemiological approaches, strengthened surveillance systems, rational antimicrobial use, and accelerated development of effective therapeutics and broadly protective vaccines.
